# Anxiety and Depression Disorders in Vietnamese Patients With Irritable Bowel Syndrome: A Cross‐Sectional Clinic‐Based Study

**DOI:** 10.1002/jgh3.70116

**Published:** 2025-02-13

**Authors:** Trung Nam Phan, Quoc Khanh Tran, Xuan Long Truong, Thi Huyen Thuong Nguyen

**Affiliations:** ^1^ Gastroenterology and Endoscopy Center Hospital of University of Medicine and Pharmacy, Hue University Hue Vietnam

**Keywords:** anxiety, depression, HADS, IBS, Vietnam

## Abstract

**Background:**

Irritable bowel syndrome (IBS) is a prevalent functional gastrointestinal disorder. Growing evidence suggests a significant association between IBS and psychological problems, such as anxiety and depression. This study was conducted to assess the prevalence of anxiety and depression in Vietnamese patients diagnosed with IBS according to Rome IV criteria.

**Methods:**

This cross‐sectional study recruited 186 consecutive patients who underwent outpatient clinic visits and colonoscopy for gastrointestinal symptoms. IBS diagnosis was established using the validated Rome IV criteria. Anxiety and depression were assessed using a validated Vietnamese version of the Hospital Anxiety and Depression Scale (HADS).

**Results:**

The mean age of IBS patients was 49.5 ± 12.0 years, with females comprising 53.8%. IBS‐M was the most prevalent subtype (39.8%), followed by IBS‐C (39.2%) and IBS‐D (21.0%). Using the HADS cut‐off of ≥ 11 points for probable anxiety and depression, the prevalence was 21.0% and 11.8%, respectively. Expanding the criterion to a HADS of ≥ 8, indicating significant symptoms, increased the prevalence to 55.9% for anxiety and 40.8% for depression disorders. Patients with IBS‐C, IBS‐D, or IBS‐M exhibited a significantly higher risk of depressive disorders compared to those without IBS, with odds ratios of 4.261, 7.013, and 6.585, respectively (*p* < 0.001). Additionally, men were less likely than women to experience depressive disorders.

**Conclusion:**

The findings revealed a high prevalence of depression and anxiety disorders among Vietnamese patients with IBS. Those with the IBS‐M or IBS‐D subtypes and a greater number of gastrointestinal symptoms were more likely to experience higher levels of depression and anxiety, particularly women.

## 1 Introduction

Irritable Bowel Syndrome (IBS) is a chronic functional intestinal disorder characterized by abdominal pain and changes in bowel habits [1]. IBS is prevalent across all age groups, impacting patients' quality of life and generating significant healthcare costs [2, 3, 4]. Currently, diagnosis of IBS primarily relies on the Rome IV criteria established in 2016 [1, 5]. The etiology of IBS is complex, involving the gut‐brain axis and the interplay between psychological and IBS symptoms [6]. The perception of pain is influenced by the emotional mechanism, therefore, IBS symptoms can also be affected by the psychological state [7]. Several published studies have mapped IBSs relationship with psychological disorders, especially, anxiety and depression [8, 9, 10]. Furthermore, recent research on human genome showed IBS may share a more similar genetic basis with psychological comorbidity which could explain genetic overlap with each other [11]. Notably, It demonstrates a relatively high genetic correlation between IBS and major anxiety and depression disorders [12].

The Hospital Anxiety and Depression Scale (HADS) is a widely used screening tool for anxiety and depression disorders in patients with chronic diseases in hospital settings. Its advantages include brevity, ease of understanding, and the simultaneous assessment of both anxiety and depression disorders [13].

Studies in Vietnam reported a range of 10.9%–17.4% for the prevalence of IBS diagnosed using the Rome criteria [14, 15]. However, researches on the association between psychological disorders and IBS in Vietnamese individuals remains limited. This study aims to utilize the HADS to investigate anxiety and depression status among patients with IBS.

## 2 Methods

### 2.1 Study Population

This cross‐sectional study investigated the prevalence and characteristics of IBS in adult patients with digestive symtomps. Data were collected from June 2021 to August 2022 using a validated Vietnamese version of the IBS questionnaire and a research form administered to 523 patients aged ≥ 18 years who visited outpatient clinics and underwent colonoscopy at Hue University of Medicine and Pharmacy Hospital and Dong Hoi Friendship Hospital.

### 2.2 Data Collection

Participants meeting the Rome IV criteria for IBS [1], as assessed by the IBS questionnaire, were eligible for inclusion (*n* = 186). Individuals with a history of melena, hematochezia, fever, significant unintentional weight loss, anemia, previous gastrointestinal surgery, familial history of colorectal cancer or inflammatory bowel disease were excluded.

IBS subtypes were classified based on stool form during episodes of abdominal pain using the Bristol Stool Form Chart (BSFC) [16]. Predominant bowel habits are based on stool form on days with at least one abnormal bowel movement, three main subtypes:

– IBS‐C (Constipation‐predominant): BSFC type 1 or 2% > 25% and type 6 or 7% < 25% of bowel movements.
– IBS‐D (Diarrhea‐predominant): BSFC type 6 or 7% > 25% and type 1 or 2% < 25% of bowel movements.
– IBS‐M (Mix): BSFC types 1, 2% > 25% and types 6, 7% > 25% of bowel movements.

All participants completed a validated Vietnamese version of the HADS. This instrument comprises 14 questions, with seven items for each subscale of anxiety or depression. Each subscale's total score ranges from 0 to 21, with the following interpretation: 0–7: Normal range, 8–10: Possible anxiety or depression, 11–21: Probable anxiety or depression, ≥ 8: Significant anxiety or depression [17].

### 2.3 Statistical Analysis

Descriptive statistics were performed on the participants' characteristic variables using SPSS 20.0. Continous variables such as age, were expressed as mean ± standard deviation (SD). Categorical variables such as gender and cases of anxiety and depression were expressed as proportion and percentages. Comparison of continous and categorical variables between groups was performed using anova and *χ* ^2^ test, respectively. A two‐sided test was used and *p* < 0.05 was considered statistically significant.

### 2.4 Ethics approval

This study was approved by the Ethics Committee of the University of Medicine and Pharmacy, Hue University, Vietnam, under protocol number H2021‐343. All participants provided written informed consent after receiving a detailed explanation of the study's purpose, procedures, and potential risks and enefits. They were assured of anonymity and the right to withdraw rom the study at any time.

## 3 Results

**FIGURE 1:**
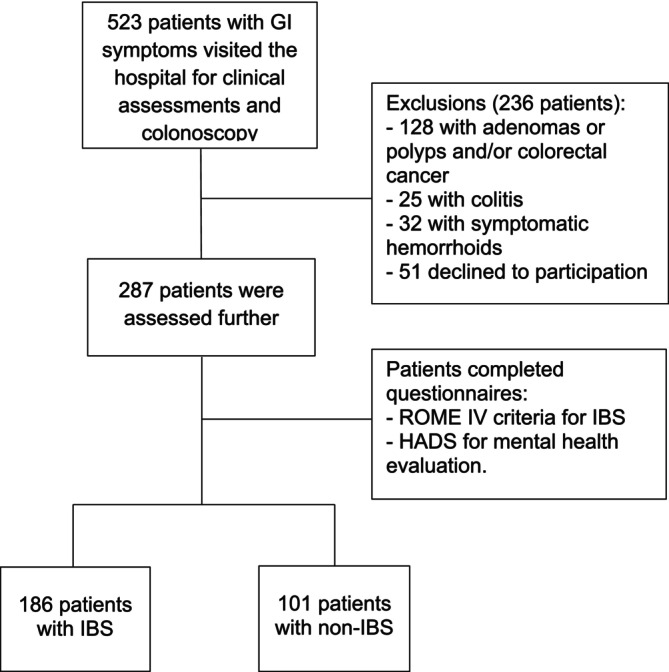
Recruitment and flow through the study.

A study was conducted on 523 adults aged 18 years or older with symptoms of gastrointestinal disorders who visited our hospitals and flow of participant through the study in detail in the Figure 1. The study found that 186 patients met the Rome IV criteria for irritable bowel syndrome (IBS), accounting for 35.5%. All of these patients underwent colonoscopy and basic blood tests to rule out organic diseases. The common characteristics of the 186 IBS patients (Table 1) were an average age of 49.5 ± 12.0 years, ranging from 18 to 75 years. The age group 30–49 had the highest proportion (46.7%), and females accounted for 53.8% (Table 2). Other gastrointestinal symptoms in IBS patients showed in detail in Table 3.

**TABLE 1:** Age distribution in patients with IBS.

Age in years	Number of patients	Percentage
< 30	28	15.1%
30–39	38	20.4%
40–49	49	26.3%
50–59	34	18.3%
60–69	26	14.0%
≥ 70	11	5.9%
**Total**	**186**	**100%**

**TABLE 2:** IBS subtypes according to sex.

IBS subtypes	Male		Female		Total *n* (%)
	*n*	%	*n*	%	
IBS‐D	21	53.8%	18	46.2%	39 (21.0)
IBS‐C	38	52.1%	35	47.9%	73 (39.2)
IBS‐M	27	36.5%	47	63.5%	74 (39.8)
**IBS Total**	**86**	**46.2%**	**100**	**53.8%**	**186 (100)**

**TABLE 3:** Other gastrointestinal symptoms in IBS patients.

Gastrointestinal symptoms	Number (%)
Bloating	102 (35.5)
Belching	54 (18.8)
Tenesmus	70 (24.4)
Nausea	22 (7.7)
Early satiety	63 (22.0)
Postprandial fullness	143 (49.8)

The clinical subtypes of 186 IBS patients (Table 2) showed that IBS‐M had the highest proportion, accounting for 39.8%. IBS‐C and IBS‐D followed with prevalence of 39.2% and 21.0%, respectively. In particular, the rate of females with IBS‐M was significantly higher than that of males (63.5% vs. 36.5%; *p* < 0.05).

### 3.1 Anxiety and Depression in IBS Patients According to the HADS Scale

Analysis of the anxiety and depression status in these IBS patients according to the HADS (Table 4) showed that the rates of probable anxiety and depression were 21.0% and 11.8%, respectively; the rates of possible anxiety and depression were 34.9% and 29.0%, respectively. Females have a higher prevalence of anxiety and depressive disorders than males.

**TABLE 4:** Anxiety and depression status in IBS patients according to the HADS.

HADS (range score)		Male (*n* = 86)	Female (*n* = 100)	Total (*n* = 186)
Anxiety status	Normal (0–7)	43 (50.0%)	39 (39.0%)	82 (44.1%)
	Possible anxiety (8–10)	28 (32.6%)	37 (37.0%)	65 (34.9%)
	Probable anxiety (11–21)	15 (17.4%)	24 (24.0%)	39 (21.0%)
Depression status	Normal (0–7)	59 (68.6%)	51 (51.0%)	110 (59.2%)
	Possible depression (8–10)	19 (22.1%)	35 (35.0%)	54 (29.0%)
	Probable depression (11–21)	8 (9.3%)	14 (14.0%)	22 (11.8%)

Association between anxiety and depression in IBS patients with clinical subtypes (Tables 5 and 6). The results of the study showed that the highest rate of probable anxiety was observed in IBS‐M patients, followed by IBS‐D and IBS‐C (Table 5). However, there was no significant difference between the patient groups according to clinical subtypes when assessing for significant anxiety symptoms (HADS ≥ 8). Similarly, the highest rate of probable depression was observed in IBS‐M patients, followed by IBS‐C and IBS‐D (Table 6). When analyzing the group of patients with possible depression symptoms, IBS‐D patients had the highest rate. However, the difference between the groups was not statistically significant.

**TABLE 5:** The relationship between anxiety status and the IBS‐subtypes.

IBS‐subtypes	Probable anxiety		Possible anxiety		Normal		Total (*n*)
	*n*	%	*n*	%	*n*	%	
IBS‐D	8	20.5%	13	33.3%	18	46.2%	39
IBS‐C	11	15.1%	28	38.4%	34	46.6%	73
IBS‐M	20	27.0%	24	32.4%	30	40.5%	74
**Total**	**39**	**21.0%**	**65**	**34.9%**	**82**	**44.1%**	**186**

**TABLE 6:** The relationship between depression status and the IBS‐subtypes.

IBS‐subtypes	Probable depression		Possible depression		Normal		Total (*n*)
	*n*	%	*n*	%	*n*	%	
IBS‐D	3	7.7%	15	38.5%	21	53.8%	39
IBS‐C	6	8.2%	19	26.0%	48	65.8%	73
IBS‐M	13	17.6%	20	27.0%	41	55.4%	74
**Total**	**22**	**11.8%**	**54**	**29.0%**	**110**	**59.2%**	**186**

Table 7 highlights several risk factors for depressive disorders (significant symptoms) in individuals with IBS. Men were less likely than women to experience depressive disorders. Patients with IBS‐C, IBS‐D, or IBS‐M exhibited a significantly higher risk of depressive disorders compared to those without IBS, with odds ratios of 4.261, 7.013, and 6.585, respectively (*p* < 0.001). Additionally, the presence of more than three clinical symptoms, including upper gastrointestinal symptoms, was associated with an increased risk of depressive disorders.

**TABLE 7:** Risk factors for depressive disorders in IBS patients.

Clinical characteristics of IBS		Depressive disorder (Significant symptoms, HADS ≥ 8)		
		OR	95% CI	*p*
Sex	Male	0.510	0.30–0.85	0.011
	Female	1		
IBS‐subtypes	IBS‐C	4.261	1.932–9.398	< 0.001
	IBS‐D	7.013	2.886–17.041	< 0.001
	IBS‐M	6.585	3.031–14.307	< 0.001
	Non‐IBS	1		
More than three gastrointestinal symptoms		16.139	7.996–32.575	< 0.001

## 4 Discussion

This is one of the first studies in Vietnam to evaluate anxiety and depression disorders in patients with IBS. The subjects of our study were people with gastrointestinal symptoms who came to the hospital for consultation. After excluding organic causes, patients were diagnosed with IBS using the standardized Rome IV questionnaire. As a result, the prevalence of IBS in our study is relatively high compared to other studies with subjects from specific populations or communities [14, 15]. The mean age of the subjects in our study was also relatively high, with most subjects in the middle‐aged group of 30–49 years. Our study results are consistent with many studies on IBS, showing that females have a higher prevalence than males [2, 18]. When analyzed by IBS subtype, the results showed that IBS‐M had the highest prevalence, followed by IBS‐C and IBS‐D, and women had a higher prevalence than men with statistical significance in the IBS‐M group. Some previous studies on the distribution of clinical subtypes have shown that IBS‐M has a higher prevalence than IBS‐D and IBS‐C [10]. By contrast, studies have inconsistent results which can be explained by the specific nature of IBS and the differences in culture, geography, study population, and diagnostic criteria used [19, 20].

The current study, we used the HADS to assess anxiety and depression disorders in patients with IBS. This tool is considered to be suitable for patients who come for consultation or treatment at the hospital with the advantage of excluding psychosomatic symptoms that often overlap with IBS digestive symptoms [21]. We divided into three main groups from the HADS assessment results as follows: normal (< 8 points), possible (8–10), probable (11–21) [17]. This study found that the prevalence of probable anxiety and depressive disorders was 21.0% and 11.8%, respectively, with a significantly higher prevalence in women. When using a cut‐off score of ≥ 8 for significant anxiety or depression, the prevalence rose to 55.9% and 40.8%, respectively. Analyzing the relationship between anxiety and depressive disorders and IBS clinical subtypes revealed that the IBS‐M group had the highest prevalence of probable anxiety or depression. Furthermore, our analysis of factors related to depressive disorders revealed that men have a lower risk of depressive disorders compared to women. Individuals with irritable bowel syndrome, including the clinical subtypes IBS‐C, IBS‐D, and IBS‐M, showed a significantly higher risk of depressive disorders than those without IBS, with odds ratios of 4.261, 7.013, and 6.585, respectively. These findings align with the meta‐analysis by Hu et al., which reported that IBS‐D and IBS‐M is strongly associated with an increased risk of depressive disorders in IBS patients [10]. Additionally, it has been established that IBS patients are at a higher risk of depressive disorders compared to individuals without IBS [8]. Interestingly, this study revealed that the presence of more than three clinical symptoms, including upper gastrointestinal symptoms, was associated with an increased risk of depressive disorders in IBS patients. Numerous studies have highlighted the high prevalence of psychological disorders in patients with functional gastrointestinal disorders, particularly IBS, with women being disproportionately affected [18, 22]. A recent study has demonstrated the direct relationship between the central and enteric nervous systems and their interaction, the brain‐gut axis [11]. This shows the causal relationship between the two central nervous and digestive systems whenever there is a functional disorder of one of the two systems, and as a result, IBS and anxiety or depression disorders will appear simultaneously in these patients [6, 12]. This is the factor that makes it difficult in clinical practice in some patients who do not respond to diet changes and medication. It is important to pay attention to mental disorders in patients with IBS to have an effective treatment plan with the personalized principle that has been recommended in many recent consensuses [23, 24].

Our study has several limitations. As the study was hospital‐based, its findings may not be generalizable to patients with IBS in the community who do not consult for their symptoms. Additionally, the data were cross‐sectional, and a formal psychiatric evaluation was not conducted to validate the results obtained through the HADS.

## 5 Conclusion

The findings of this study revealed a high prevalence of depression and anxiety disorders among Vietnamese patients with IBS. Those with the IBS‐M or IBS‐D subtypes and a greater number of gastrointestinal symptoms were more likely to experience higher levels of depression and anxiety, particularly women. Psychological screening and appropriate psychotherapy are crucial for effectively managing IBS patients.

## Conflicts of Interest

The authors declare no conflicts of interest.
